# Rifampicin Protects PC12 Cells from Rotenone-Induced Cytotoxicity by Activating GRP78 via PERK-eIF2α-ATF4 Pathway

**DOI:** 10.1371/journal.pone.0092110

**Published:** 2014-03-17

**Authors:** Xiuna Jing, Qiaoyun Shi, Wei Bi, Zhifen Zeng, Yanran Liang, Xia Wu, Songhua Xiao, Jun Liu, Lianhong Yang, Enxiang Tao

**Affiliations:** 1 Department of Neurology, Sun Yat-sen Memorial Hospital of Sun Yat-sen University, Guangzhou, People's Republic of China; 2 Department of Radiology, School of Medicine, Stanford University, Stanford, California, United States of America; 3 Department of Neurology, the First Affiliated Hospital of Jinan University, Guangzhou, People's Republic of China; Temple University School of Medicine, United States of America

## Abstract

Rifampicin has been proposed as a therapeutic candidate for Parkinson's disease (PD). We previously showed that rifampicin was neuroprotective in PD models in vivo and in vitro. However, the molecular mechanisms underlying are not fully elucidated. In this study, using the comprehensive proteomic analysis, we identified that the 78 kDa glucose-regulated protein (GRP78), a hallmark of the unfolded protein response (UPR), was upregulated in rifampicin-treated PC12 cells. Western blot analysis confirmed GRP78 activation. GRP78 functions cytoprotectively in stressed cells, therefore, we hypothesized that GRP78 mediated rifampicin-induced neuroprotection. Using RNA interference, we found that GRP78 gene knockdown significantly attenuated the neuroprotective effects of rifampicin. Next, we examined three UPR transducers, namely, protein kinase RNA-like endoplasmic reticulum kinase (PERK), inositol requiring kinase α (IREα) and activating transcription factor 6 (ATF 6), and how they regulated rifampicin-stimulated GRP78 expression. Our results showed that PERK, eukaryotic initiation factor 2α (eIF2α), and activating transcription factor 4 (ATF4) were activated in rifampicin-treated PC12 cells. Silencing the ATF4 gene using RNAi inhibited GRP78 stimulation. Interestingly, we did not detect significant IREα activation, X-box binding protein 1 mRNA splicing, or ATF6 cleavage up to 24 h after rifampicin treatment. Taken together, our data suggested that rifampicin induced GRP78 via the PERK-eIF2α-ATF4 pathway to protect neurons against rotenone-induced cell damage. Targeting molecules in this pathway could be a novel therapeutic approach for PD treatment.

## Introduction

Parkinson's disease (PD) is the second most common neurodegenerative disorder after Alzheimer's disease. Neuropathologically, it is characterized by the progressive loss of dopaminergic neurons within the substantia nigra pars compacta of the midbrain [1]. Current PD treatments are focused on symptomatic relief, which have risks of causing severe side effects and fail to prevent or delay the progression of the disease [2]. Therefore, searching for novel therapies to reduce the loss of dopaminergic neurons will shed new light on PD treatments.

Rifampicin is an antibiotic that is widely used for tuberculosis and leprosy. It has been proposed to treat Parkinson's disease [3]. Reports using PD models have demonstrated that it is neuroprotective in vivo [4] and in vitro [5]. In line with this, our previous study showed that rifampicin protected PC12 cells against 1-methyl-4-phenylpyridinium (MPP+)-induced apoptosis [6]. Pre-treatment with rifampicin decreased rotenone-induced neurotoxicity in rats [7]. However, the molecular mechanisms underlying the neuroprotection of rifampicin remain unknown.

In the present study, we performed a comprehensive proteomic analysis to explore the mechanisms by which rifampicin elicited protective cellular responses. The expression of the glucose-regulated protein 78 (GRP78) was significantly increased in rifampicin-treated PC12 cells. This result was confirmed by Western blot analysis. Gene silencing using RNA interference verified the mediation of GRP78 in rifampicin-induced neuroprotection.

GRP78, also known as Bip, is a chaperone protein localized in the endoplasmic reticulum (ER) and plays an important role in cytoprotection and cell survival [8], [9]. GRP78 is the hallmark of unfolded protein response (UPR) [10]. UPR is a cellular defense system in response to the accumulation of misfolded proteins under ER stress [11]. UPR induces the expression of GRP78 by activating ER-resident transmembrane proteins, including the activated pancreatic ER kinase-like ER kinase (PERK), inositol requiring kinase α (IREα) and activating transcription factor 6 (ATF 6) [12]. Increasing evidence has suggested that GRP78 activation prevents neurons from apoptosis [13], [14], [15]. Therefore, we hypothesized that rifampicin protected PC12 cells against rotenone-induced cytotoxicity by regulating the GRP78 gene expression. We also investigated the signaling pathways through which rifampicin stimulated GRP78. Our study was aimed to explore potential novel therapeutic targets for PD treatment.

## Methods

### Materials

Rifampicin, Rotenone, dimethyl sulfoxide (DMSO), 3-(4,5-Dimethylthiazol-2-yl)-2,5-diphenyltetrazolium bromide (MTT), 4′,6-diamidino-2-phenylindole (DAPI) and thapsigargin (Tg) were purchased from Sigma (St. Louis, MO, USA). Rifampicin was dissolved in less than 0.1% of DMSO solution. RPMI medium 1640, fetal horse serum (FCS), fetal bovine serum (FBS), penicillin, streptomycin, and other tissue culture reagents were purchased from Gibco (Grand Island, NY, USA). Antibodies against PERK(sc-13073), p-PERK(sc-32577), ATF6, and beta-actin were from Santa Cruz Biotechnology (Santa Cruz, CA, USA). Antibodies against GRP78, p-eIF2α, eIF2α and ATF4 were from Cell Signaling (Beverly, MA, USA). Antibodies against p-IREα were from Abcam (Hong Kong, China).

### Cell Culture

PC12 cells were purchased from the Cell Center of the Institute of Basic Medical Science Research (Chinese Academy of Medical Sciences, China). Cells were cultivated in RPMI medium 1640 supplemented with 10% heat-inactivated fetal horse serum, 5% heat-inactivated fetal bovine serum, 100 U/mL penicillin, and 100 μg/mL streptomycin. Cells were kept at 37 °C in a humidified atmosphere with 5% CO_2_. Growth medium was changed three times a week. Unless indicated otherwise, prior to the experimental investigation, PC12 cells were differentiated by adding nerve growth factor (NGF) at 50 ng/mL every other day for 6 days, followed by rifampicin treatment at 150 μM for 24 h. In GRP78 gene silencing study, after differentiation and siRNAs treatment, PC12 cells were incubated with 150 μM rifampicin for 2 h followed by 1 μM rotenone for 24 h.

### Cell Viability Assay

PC12 cells were seeded at a density of 1×10^4^ cells/well in 96 well plates, and the cell viability was determined by the conventional MTT assay. Briefly, cells were treated with the MTT solution for 4 h at 37 °C. Then, the medium was removed and 150 μL of DMSO was added to each well. The formazan dye crystals were solubilized on the shaker for 15 min, and the absorbance at 595 nm was measured using a microplate reader (Bio-Rad, Hercules, CA, USA). Cell viability was determined by comparing the number of viable cells to that of untreated controls, in which the viability was defined as 100%.

### DAPI staining

After treatment, cells were washed with PBS and fixed with 4% formaldehyde for 30 min at room temperature. Following incubation with Triton X-100 0.2% in PBS for 5 min, PC12 cells were incubated with 4′,6-diamidino-2-phenylindole (DAPI; 1 μg/ml) for 5 min at room temperature.

### 2-DE and Image Analysis

PC12 cells were treated with or without rifampicin (150 μM) for 24 h. After treatment, cells were washed three times with ice-cold washing buffer (10 mM Tris-HCl, 250 mM sucrose, pH 7.0), collected in clean 1.5 ml eppendorf tubes. Lysis buffer [7 Murea, 2 M thiourea, 4% CHAPS (w/v), 1% dithiothreitol (DTT), 2% immobilized pH gradients (IPG) (v/v), pH 3–10 NL] was added, and samples were centrifuged at 13,200 g for 30 min at 4 °C. The supernatant was subjected to 2-DE using an Amersham Biosciences IPGphor IEF System and Hoefer SE 600 (GE healthcare, Uppsala, Sweden) electrophoresis units (13 cm), according to manufacturer's instructions and a previously described protocol [16]. Protein lysates and 2-DE gels were processed in parallel. Protein concentrations were determined using the Bradford assay. After 2-DE, the gels underwent silver nitrate staining according to a previously described protocol [17], followed by scanning using an Image Scanner (GE Healthcare). The images were analyzed using the Image Master 2D Platinum (GE Healthcare).

### MALDI-TOF-MS and Database Search

Only protein spots that were differentially expressed in at least three independent experiments were analyzed by MALDI-TOF-MS. Protein spots were excised from the silver-stained gels and transferred into the siliconized 1.5 ml eppendorf tubes. Tryptic in-gel digestion was performed as previously reported with slight modifications [16]. Molecular mass analysis of the tryptic peptides was performed using an ABI 4800 plus a MALDI-TOF-MS mass spectrometer (Applied Biosystems, Foster City, CA, USA). Spectra were interpreted and processed using the Global Protein Server Workstation (V3.6, Applied Biosystems) via the internal MASCOT search engine (V2.1, Matrix Science, London, UK) to analyze MALDI-TOF-MS and MS/MS data. Based on combined MALDI-TOF-MS and MS/MS spectra, MASCOT protein scores of greater than 65 were considered statistically significant (p<0.05). It was also accepted when the individual MS/MS spectrum had the best ion score that was statistically significant (p<0.05). Searching was performed against the IPI mouse database (V3.36) with the following parameters: the enzyme trypsin with one missed cleavage was allowed; variable modifications included acetamidation of cysteine and oxidation of methionine; peptide mass tolerance was set to 50 ppm and fragment ion mass tolerance was set to 0.2 Da; only monoisotopic masses were included in the search.

### Western Blot Analysis

After treatment, PC12 cells were harvested for western blot analysis. Cell pellets were briefly lysed in RIPA buffer [1 mM ethylenediaminetetraacetic acid (EDTA), 150 mM NaCl, 1% igepal, 0.1% SDS, 0.5% sodium deoxycholate, 50 mM Tris–HCl, pH 8.0]. Equal amounts of proteins (50 μg) were separated by 10% sodium dodecyl sulfate polyacrylamide gel electrophoresis (SDS-PAGE), transferred to polyvinylidene fluoride (PVDF) membranes (Millipore Corp, MA, USA), blocked with 5% nonfat milk for 2 h, and incubated overnight at 4 °C with primary antibodies at a dilution of 1:1000 in blocking buffer. The next day, the membrane was washed by TBST three times, 10 min each, and incubated with the corresponding secondary antibodies (1:4000) that were horseradish peroxidase-conjugated for 1 h at room temperature.

Analysis was detected using the Syngene G:BOX Chemi XT4 fluorescence and chemiluminescence gel imaging system (Cambridge, UK).

### RNA extraction and RT-PCR

PC12 cells were treated with rifampicin for various periods of time (3 to 24 h). In positive controls, PC12 cells were incubated with Tg at 1 μM for 6 h. Total RNA was isolated using Trizol reagent (Invitrogen, Groningen, NL). cDNA was synthesized using the Superscript III First strand synthesis Kit (Invitrogen). To evaluate relative expression levels of XBP1u/XBP1s, RT-PCR analysis was performed using PCR SuperMix (Invitrogen). XBP1 primer sequences were as follows: 5′-GGCGGCCCCCAAAGTGCTAC-3′ (Forward) and 5′-CCCGGAACCATGAGCGGCAG-3′ (Reverse). β-actin was used as a loading control, with primers as follows: 5′-GCGTCCACCCGCGAGTACAA-3′ (Forward) and 5′-CGACGACGAGCGCAGCGATA-3′ (Reverse). PCR products were analyzed on a 3% agarose gel. Gene expression was quantified using ImageJ 1.45 s.

### RNA Interference and Transfection

Gene silencing was performed using siRNAs. GRP78-specific siRNAs were synthesized by Shanghai GenePharma (Shanghai, China) with the sense strand sequence of 5′-GAGGCGUAUUUGGGAAAGATT-3′.

The scrambled siRNA has the sense sequence of 5′-UUCUCCGAACGUGUCACGUTT-3′. To efficiently knock down ATF4, we used a pool of four different siRNAs (ON-TARGET plus SMART pool Rat ATF4; Thermo Scientific Dharmacon) to target rat ATF4 mRNA. Control cultures were incubated with non-targeting siRNAs (ON-TARGET plus non-targeting siRNA#1; Thermo Scientific Dharmacon).

Transfection of siRNAs was carried out using Lipofectamine™ 2000 (Invitrogen, Grand Island, NY, USA), according to the manufacturer's instructions. Briefly, siRNA and Lipofectamine™ 2000 reagent were mixed in Opti-MEM medium (Invitrogen) and incubated for 30 min at room temperature to allow the complex formation. Cells were washed with Opti-MEM medium, and the transfection mixture was added. 6 h after transfection, cells were washed and cultured for 24 h in complete medium containing 10% FCS and 5% FBS. The silencing efficacy was evaluated by western blotting.

### Statistical Analysis

All data were presented as the mean ± standard error of the mean (SEM) derived from three or more independent experiments. Comparison between groups was made by one-way analysis of variance (ANOVA) followed by an appropriate post-hoc test to analyze the difference. A value of p<0.05 was deemed to be statistically significant.

## Results

### GRP78 mediated rifampicin-induced neuroprotection

**Figure 1:**
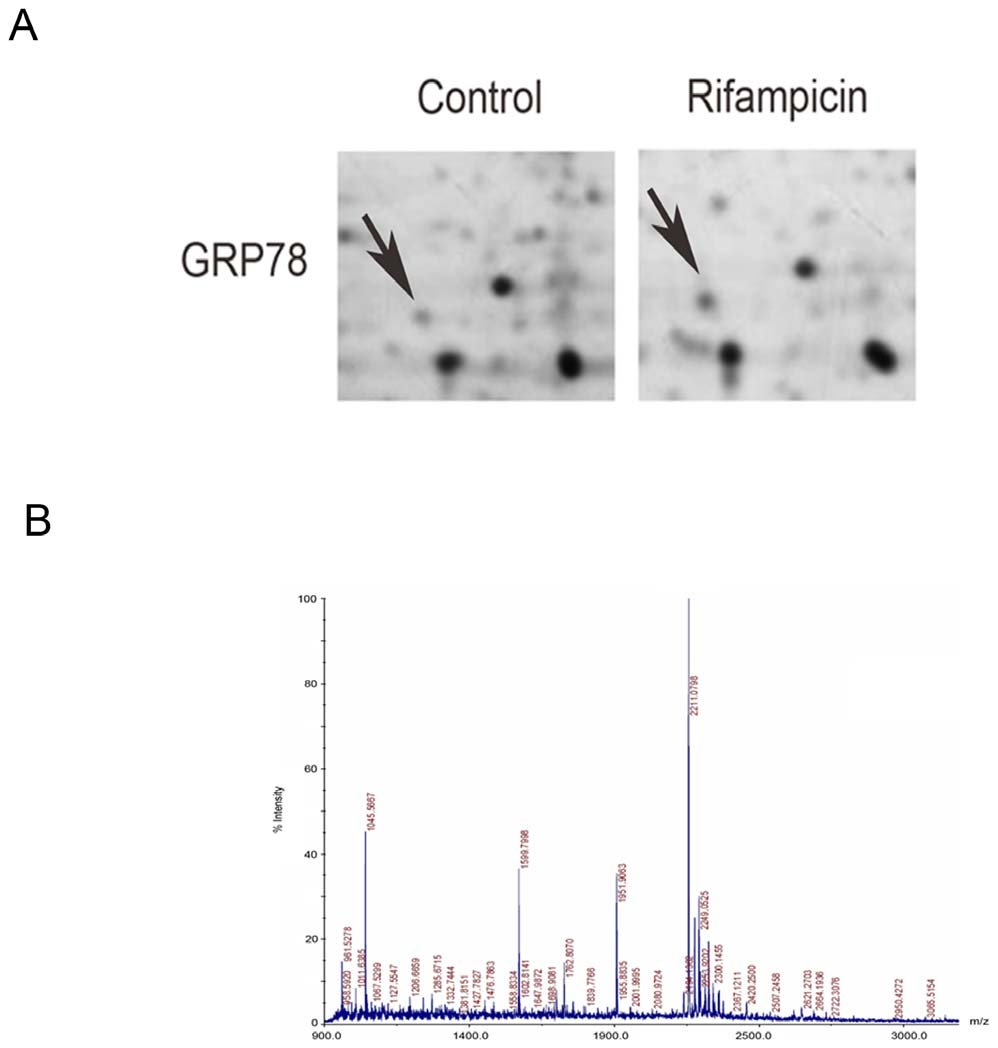
2D-DIGE gel images of proteins isolated from rifampicin-treated PC12 cells. (A) Arrows indicate proteins that were differentially expressed in PC12 cells treated with or without rifampicin. (B) Representative peptide mass fingerprint spectra generated by MALDI-TOF-MS. The x-axis indicates the mass-to-charge ratio, m/z. The y-axis indicates the relative abundance. Peptide masses are labeled and the corresponding m/z is annotated.

To elucidate the underlying mechanisms by which rifampicin improves neuron survivals, we performed a comprehensive proteomic analysis to identify molecules mediating the process. After matching with two-dimensional electrophoresis (2-DE) maps, 15 protein spots were selected for further investigation by matrix-assisted laser desorption/ionization time-of-flight mass spectrometry (MALDI-TOF-MS). Among them, GRP78 was identified (Figure 1).

**Figure 2:**
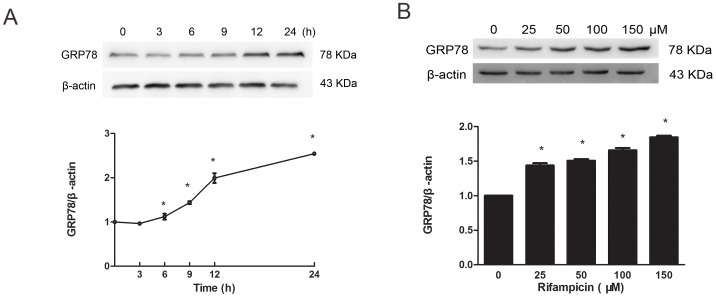
Rifampicin induced a time- and dose-dependent GRP78 activation. (A) Cells were treated with rifampicin at 150 μM for indicated periods of time. Western blot analysis showed that rifampicin significantly upregulated GRP78 protein expression at 6 h post-treatment. Prolonged rifampicin incubation further enhanced GRP78 induction up to 24 h after treatment. (B) Cells were treated with rifampicin at indicated concentrations, followed by western blot analysis to measure GRP78 activation. Rifampicin induced a dose-dependent upregulation of GRP78 expression. Protein expression was relative to control cells, in which GRP78 expression was deemed to be 1. Data present mean ± SEM of three independent experiments. *p<0.05 compared with control groups.

**Figure 3:**
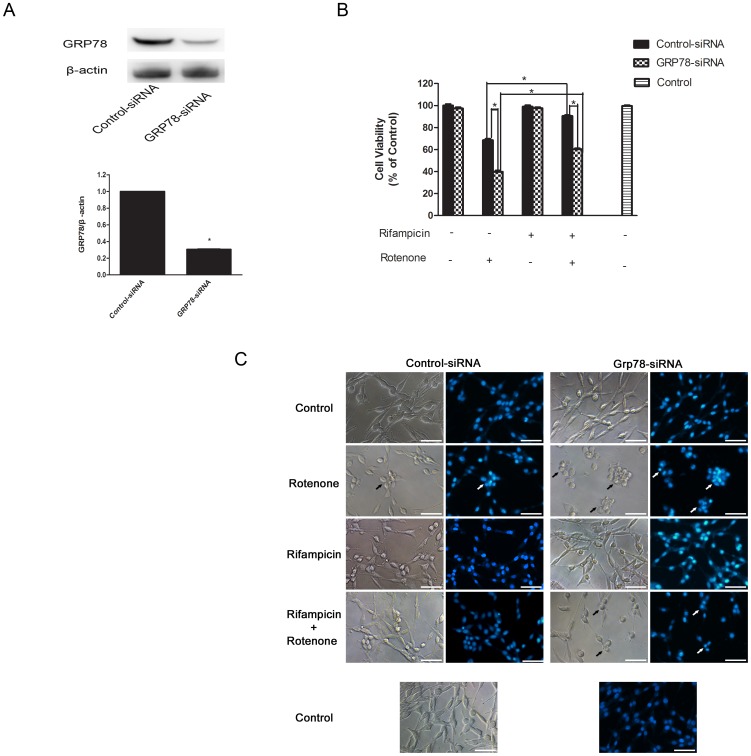
Rifampicin-induced neuroprotection was GRP78-dependent. PC12 cells were transfected with GRP78-specific siRNAs or control siRNAs for 24 h. After that, cells were treated with or without 150 μM rifampicin for 2 h, followed by 1 μM rotenone for 24 h. (A) Western blot analysis verified the efficient gene silencing of GRP78. (B) After the above treatment, cell viability was measured and presented as the relative viability (% control). (C) Morphological evaluation of PC12 cells under the above-mentioned treatment by light microscopic observation and DAPI staining. The apoptotic cells were marked with arrows. Scale bar = 25 μm. (B–C) GRP78 gene silencing significantly exacerbated rotenone-triggered neuron injury, with or without the presence of rifampicin. Neither GRP78-specific nor control siRNAs decreased cell viability. Data present mean ± SEM of three independent experiments. *p<0.05.

To verify the results of the proteomic analysis, western blot analysis was conducted. As shown in Figure 2A, rifampicin induced a significant increase of GRP78 protein expression in PC12 cells at 6 hours (h) post-incubation (p<0.05). Prolonged rifampicin incubation further enhanced GRP78 induction up to 24 h after treatment (p<0.05). When incubated with increasing concentrations of rifampicin ranging from 25 to 150 μM, PC12 cells showed a dose-dependent induction of GRP78 (p<0.05, Figure 2B). The greatest activation was observed at 150 μM of rifampicin, which was not cytotoxic to PC12 cells (p>0.05, Figure 3B).

### Rifampicin-induced neuroprotection was GRP78-dependent

To test whether rifampicin-induced neuroprotection is GRP78-dependent, we used RNAi to knock down GRP78 and then evaluated cell survival and cell morphology after rifampicin pre-incubation in PC12 cells that were exposed to rotenone. Gene silencing efficacy was assessed by western blot analysis, which demonstrated 70% reduction of GRP78 expression, compared with cells transfected with the control small interference RNA (siRNA) (p<0.05, Figure 3A).

Of note, as shown in Figure 3B, GRP78-specific siRNA transfection exacerbated cell injury triggered by rotenone, with or without presence of rifampicin. Moreover, the cytoprotective effect of rifampicin on PC12 cells was significantly inhibited by GRP78 siRNA.

In addition, following different treatment we observed morphology of PC12 cells and stained them with DAPI, the specific DNA stain used to assess changes in nuclear structure, to further estimate neurotoxicity. The changes of cell morphology and nuclear structure further implied the important role of GRP78 in rifampicin-induced neuroprotection (Figure 3C). Neither GRP78 targeting siRNA nor control siRNA affected the viability (Figure 3B) and morphology of PC12 cells (Figure 3C), indicating that the decreased cell viability was specific to GRP78 gene knockdown. These results indicated that rifampicin increases cell viability of rotenone-exposed PC12 cells through GRP78 upregulation.

### The PERK-eIF2α-ATF4 pathway regulated rifampicin-induced GRP78 activation

Since GRP78 is under the transcriptional control of the UPR, we were prompted to investigate the UPR pathways in rifampicin-treated PC12 cells, including the PERK-eIF2α-ATF4, IREα-XBP1, and ATF6 pathways.

**Figure 4:**
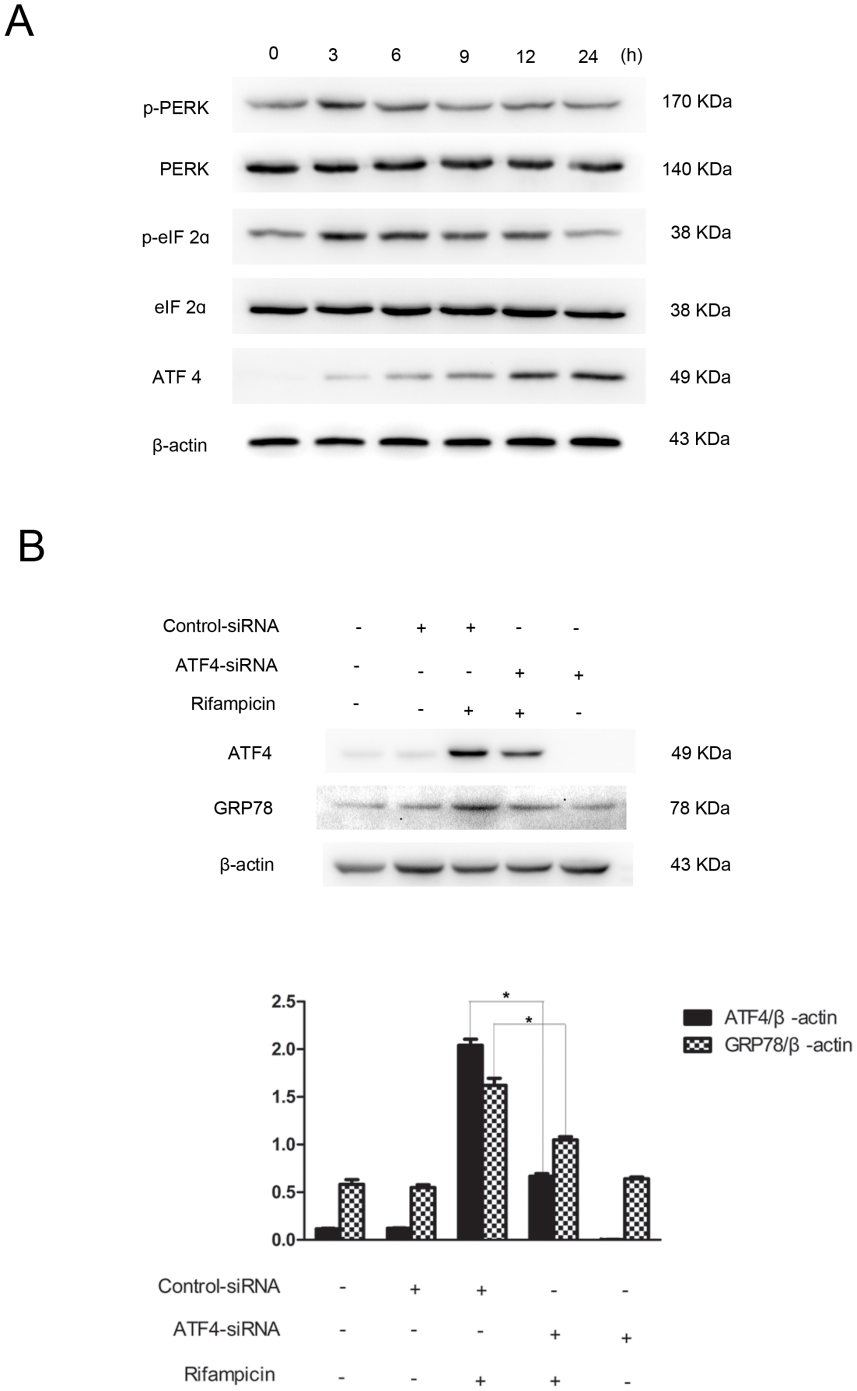
The PERK-eIF2α-ATF4 pathway regulated rifampicin-induced GRP78 activation. (A) PC12 cells were treated with 150 μM rifampicin for indicated lengths of time, ranging from 3 to 24 h. Cell lysates were analyzed by western blotting using antibodies against phosphorylated PERK (p-PERK), PERK, phosphorylated eIF2a (p- eIF2a), eIF2a, ATF4 and β-actin. Rifampicin induced a transient PERK phosphorylation at 3 h post-incubation. eIF2a was activated at 3 h post-treatment, which persisted for 9 h and started to decline thereafter. ATF4 was activated at 6 h post-treatment, which persisted up to 24 h post-treatment. (B) PC12 cells were transfected with ATF4-specific siRNAs or control siRNAs for 24 h, followed by rifampicin incubation at 150 μM for 24 h. Western blot analysis showed that ATF4 gene silencing reduced GRP78 protein expression. Data present mean ± SEM of three independent experiments. *p<0.05.

In the PERK-eIF2α-ATF4 pathway, PERK phosphorylates eIF2α, which activates ATF4. ATF4 binds to the promoter of the GRP78 gene to induce GRP78 expression [18]. In this investigation, we discovered the activation of PERK, eIF2α, and ATF4 proteins in response to rifampicin treatment (Figure 4A). Shortly after incubation, rifampicin induced a transient PERK activation, with the maximal stimulation at 3 h post-treatment. eIF2α was simultaneously activated at 3 h post-incubation, which persisted up to 12 h after treatment and started to decline thereafter. ATF4 was activated after prolonged rifampicin incubation at 6 h post-treatment, which was in agreement with GRP78 induction (Figure 2A). To assess whether GRP78 activation is ATF4-dependent, we knocked down ATF4 gene expression by RNAi. As shown in Figure 4B, ATF4 gene silencing inhibited the expression of GRP78, implying that GRP78 activation was regulated by ATF4 in rifampicin-treated PC12 cells. Taken together, our results indicated that the PERK-eIF2α-ATF4 signaling pathway regulated GRP78 stimulation after rifampicin treatment.

### No significant activation of the IREα-XBP1 or ATF6 pathway by rifampicin

We next studied the IREα-XBP1 and ATF6 pathways. Upon the UPR activation, phosphorylated-IREα splices X-box binding protein 1 (XBP1) mRNA to generate XBP1s, a potent transcription factor that activates ER chaperones, including GRP78 [19]. ATF6 is another UPR sensor that promotes the induction of GRP78 [20]. In stressed cells, p90 ATF6 is cleaved to its active form, p50 ATF6, which translocates to the nucleus, acting as a transcriptional factor to induce GRP78 expression.

**Figure 5:**
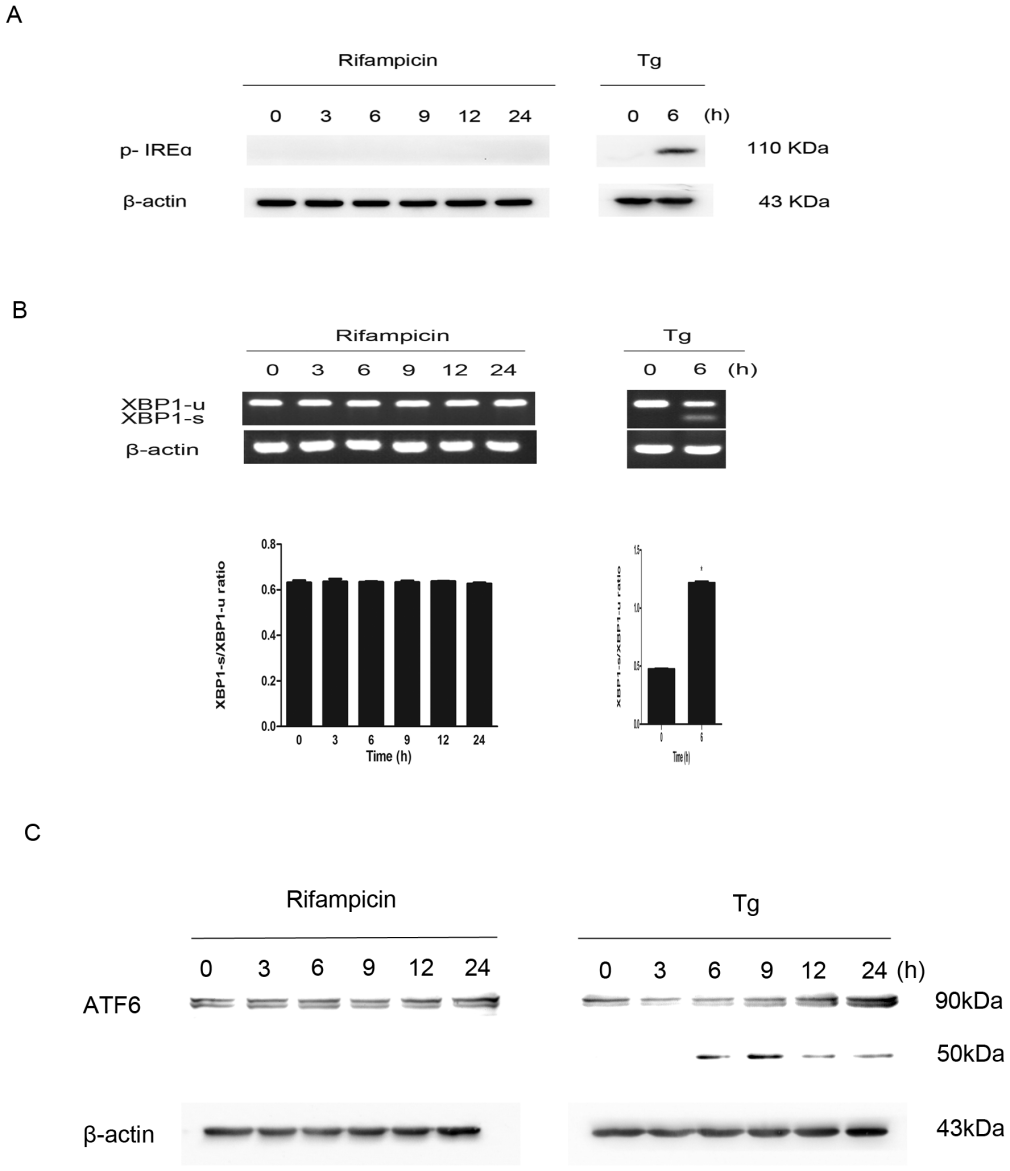
No significant activation of the IREα-XBP1 or ATF6 pathway by rifampicin. PC12 cells were incubated with 150 μM rifampicin for indicated lengths of time, ranging from 3 to 24 h. Cells treated with 1 μM Tg served as positive controls. (A) Cell lysates were subjected to western blotting using p-IREα and β-actin antibodies. Rifampicin did not stimulate IREα phosphorylation. (B) Total RNA was subjected to RT-PCR to measure XBP1u/XBP1s expression. Rifampicin did not induce splicing of XBP1 mRNA significantly. (C) Cell lysates were analyzed by western blotting using the ATF6 antibody. Rifampicin did not activate ATF6 cleavage in PC12 cells. Data present mean ± SEM of three independent experiments, with four to six replicates each.

Interestingly, when treated with rifampicin at 150 μM for various periods of time up to 24 h, PC12 cells did not show significant phosphorylation of IREα or the cleavage of ATF6, suggesting that neither the IREα-XBP1 nor ATF6 signaling pathway mediated the process. By contrast, positive control cells treated by thapsigargin (Tg) demonstrated marked activation of both pathways (Figure 5A, C). To exclude the possibility of mild activation, we measured the expression of unspliced and spliced forms of XBP1 mRNAs in rifampicin-treated PC12 cells using reverse transcription PCR (RT-PCR) analysis. Rifampicin stimulation for 3 to 24 h did not increase spliced XBP1 mRNAs significantly (p>0.05), compared with control cells treated with Tg that showed marked upregulation of spliced XBP1 mRNAs (Figure 5B).

## Discussion

The neuroprotective effect of rifampicin has been reported by various researchers. Oida Y et al. discovered that rifampicin attenuated the MPTP-induced neurotoxicity in mouse brain [4]. Ulkan Kilic et al. found that rifampicin administration significantly increased the number of surviving dopaminergic neurons after MPP+ intoxication [5]. Previously, we also showed the protective effect of rifampicin in experimental models of PD both in vivo and in vitro. We found that rifampicin increased cell survival via reducing ROS production, inhibiting α-synuclein accumulation and neuroinflammation [7], [21], [22]. However, the underlying mechanisms through which rifampicin confers neuroprotection are not fully understood.

In this investigation, using 2-DE and MALDI-TOF-MS, we successfully identified GRP78, which mediated rifampicin-induced neuroprotection in PC12 cells. The proteomic data were confirmed by western blot analysis. Gene silencing of GRP78 attenuated rifampicin-induced neuroprotection. Further investigations of UPR pathways revealed that rifampicin selectively activated the PERK-eIF2α-ATF4 pathway to regulate GRP78 stimulation. For the first time, we uncovered that rifampicin activated GRP78 via the PERK-eIF2α-ATF4 pathway to protect PC12 cells against rotenone-induced cytotoxicity.

Increasing evidence has suggested that heat shock proteins (HSPs) play a pivotal role in neurodegenerative diseases. HSPs provide a therapeutic target for neurodegenerative disorders due to the finding that upregulation of HSPs decreases the protein misfolding and aggregation in cells [23], [24], [25]. GRP78 is a member of the 70-kDa HSP family and acts as a molecular chaperone in the folding and assembly of newly synthesized proteins within the ER [26]. It is reported that GRP78 suppressed caspase activation and caspase-mediated cell death [27], suggesting it is cytoprotective. Several groups have demonstrated that GRP78 improves cell survival in vivo and in vitro [28], [29], [30], [31]. Gorbatyuk, MS et al. [32] revealed that GRP78 diminished α-synuclein-induced neurotoxicity in a rat model of Parkinson disease. In this investigation, we showed that rifampicin upregulated GRP78 expression in a time and dose dependent manner, indicating that rifampicin was a GRP78 inducer.

Next, we used RNAi to knock down GRP78 gene expression to determine whether rifampicin-induced GRP78 activation protects against cytotoxicity in rotenone-exposed PC12 cells. Our results demonstrated that cells transfected with GRP78-specific siRNA were more vulnerable to rotenone-induced cell injury than cells transfected with scramble siRNA, regardless of the presence of rifampicin. GRP78 gene silencing counteracted rifampicin-induced neuroprotection. Our findings are consistent with previous reports, which showed that downregulation of GRP78 caused cells to become sensitive and more prone to various insults [29], [33], [34]. Taken together, these data implied that the induction of GRP78 was critical to rifampicin-mediated neuroprotection.

GRP78 induction has been studied extensively as a marker for the UPR [10]. The UPR, an adaptive process, is activated in response to the disruption of ER homeostasis [35]. Once stimulated, the UPR protects against ER stress by suppressing protein translation, enhancing ER-associated degradation (ERAD), and increasing ER chaperones such as GRP78 [36]. Three UPR pathways of PERK-eIF2α-ATF4, IREα-XBP1 and ATF6 control the expression of ER chaperones. In this study, we showed that rifampicin triggered an early phosphorylation of PERK and eIF2α proteins in PC12 cells, which subsequently stimulated ATF4. ATF4 gene knockdown decreased rifampicin-induced GRP78 activation. By contrast, we did not detect IREα phosphorylation, XBP1 mRNAs splicing, or ATF6 cleavage up to 24 h after rifampicin administration. Our findings suggested that rifampicin selectively activated the PERK-eIF2α-ATF4 signaling pathway to regulate GRP78 induction.

If a GRP78 inducer is just an ER stressor such as Tg, its application as a therapeutic strategy is unlikely to be realized because it may activate several pathways of the UPR, including ER stress-induced apoptotic pathways[28]. The present study showed that the PERK-eIF2α-ATF4 pathway was activated by rifampicin to induce GRP78, neither IREα pathway or ATF6 pathway. The selective activation of one UPR pathway by rifampicin may account for its protection.

Our investigation showed that with the increase of rifampicin dosages, there was an enhancement of GRP78 activation, with the highest induction observed at 150 μM of rifampicin, which did not affect cell viability. Since GRP78 induction is a hallmark of ER stress [37], rifampicin might enhance cellular defense systems by exposing PC12 cells to a mild stress state. This phenomenon is known as preconditioning. Several lines of evidence have suggested that cells pretreated with sublethal stress could adapt to stress and increase their defense capacities to resist more severe stress [30], [38]. Here, we showed that induction of GRP78 by rifampicin “preconditioned” PC12 cells and protected them against cell injury triggered by rotenone. Although we tested rotenone-induced cytotoxicity in this study, it is possible that rifampicin and GRP78 might protect cells against other parkinsonian neurotoxins, thus, function as potential treatments for neurodegeneration because oxidative stress and ER stress have been implicated in the disease development after exposure to these toxins [39]. Nevertheless, further investigations are needed to clarify it.

To our best knowledge, we for the first time identified that rifampicin activated GRP78 to protect PC12 cells from rotenone-induced cytotoxicity. Our results provided added information to the mechanism underlying rifampicin-induced neuroprotection, particularly, signaling pathways involved in this process. Future directions include, but are not limited to, in vivo verification of GRP78 and UPR pathways in rifampicin-treated PD animal models, investigation of the functions of GRP78 proteins in ER stress and how they are regulated, identification of other potential therapeutic targets. Based on our findings, we concluded that GRP78 and PERK-eIF2α-ATF4 pathway mediated rifampicin-induced neuroprotection and their targeting could serve as a novel potential therapy for PD treatment.
